# Mechanotransduction and cell biomechanics of the intervertebral disc

**DOI:** 10.1002/jsp2.1026

**Published:** 2018-07-27

**Authors:** Bailey V. Fearing, Paula A. Hernandez, Lori A. Setton, Nadeen O. Chahine

**Affiliations:** ^1^ Department of Biomedical Engineering & Orthopedic Surgery Washington University in St. Louis St. Louis Missouri; ^2^ Department of Orthopaedic Surgery University of Texas Southwestern Dallas Texas; ^3^ Department of Orthopedic Surgery & Biomedical Engineering Columbia University New York New York

**Keywords:** biomechanics, degeneration, extracellular matrix, mechanobiology

## Abstract

Mechanical loading of the intervertebral disc (IVD) initiates cell‐mediated remodeling events that contribute to disc degeneration. Cells of the IVD, nucleus pulposus (NP) and anulus fibrosus (AF), will exhibit various responses to different mechanical stimuli which appear to be highly dependent on loading type, magnitude, duration, and anatomic zone of cell origin. Cells of the NP, the innermost region of the disc, exhibit an anabolic response to low‐moderate magnitudes of static compression, osmotic pressure, or hydrostatic pressure, while higher magnitudes promote a catabolic response marked by increased protease expression and activity. Cells of the outer AF are responsive to physical forces in a manner that depends on frequency and magnitude, as are cells of the NP, though they experience different forces, deformations, pressure, and osmotic pressure in vivo. Much remains to be understood of the mechanotransduction pathways that regulate IVD cell responses to loading, including responses to specific stimuli and also differences among cell types. There is evidence that cytoskeletal remodeling and receptor‐mediated signaling are important mechanotransduction events that can regulate downstream effects like gene expression and posttranslational biosynthesis, all of which may influence phenotype and bioactivity. These and other mechanotransduction events will be regulated by known and to‐be‐discovered cell‐matrix and cell‐cell interactions, and depend on composition of extracellular matrix ligands for cell interaction, matrix stiffness, and the phenotype of the cells themselves. Here, we present a review of the current knowledge of the role of mechanical stimuli and the impact upon the cellular response to loading and changes that occur with aging and degeneration of the IVD.

## 1 INTRODUCTION

The intervertebral disc (IVD) is a heterogeneous structure that, together with two facet joints, functions as a “three‐joint complex” to provide for load support and flexibility of the spine^1^. The IVD is subject to substantial motions due to spinal flexion, extension, and torsion, and consequently physical forces of joint loading and muscle activation. The IVD is well‐adapted to sustain these motions and loads with a composite of sub‐structures that consists of confining end plates (EPs) on the superior and inferior faces, the highly fibrous anulus fibrosus (AF) on the outer periphery, and the highly hydrated nucleus pulposus (NP) at the center. Each substructure contains large amounts of water in a dense and negatively charged extracellular matrix (ECM), but with a unique morphology and compositional differences that contribute to variable magnitudes of interstitial osmotic and fluid pressures, fluid‐flow, compressive, tensile and shear stresses, and strains. The mechanical demands of IVD loading and motion may contribute to magnitudes of each and all of these factors that could facilitate tissue failure. As an avascular, alymphatic tissue with the lowest cell density of any tissue or organ in the body^2^, the IVD has limited mechanisms by which to heal and repair so that spinal loading can promote and contribute to IVD degeneration. Some hallmarks of IVD degeneration include decreased disc height and loss of hydration that may be accompanied by loss of IVD mobility, loss of fluid pressurization, a decreased T2 MRI signal, and changes to the vertebral EPs, all of which are believed to contribute to altered IVD function.

Increasingly, evidence suggests that the cell‐mediated and biological remodeling responses to mechanical stimuli generated by spinal motions are a large contributor to degenerative and pathological changes^3^. These cell‐level responses to loading environment occur decades before disc degeneration manifests^4^, ^5^. For these reasons, studies of mechanobiology and the interactions between mechanical stimuli and biological processes are performed to understand the development of disc degeneration. Many studies have documented the biological responses of IVD cells to mechanical stimuli, but only recently have studies begun to elucidate mechanisms governing these observed mechanobiologic responses in the IVD. In this article, we present a summary of the current knowledge of IVD mechanobiology and its relevance to disc degeneration, and identify several signaling events and mechanisms that govern cellular responses to mechanical stimuli.

## 2 STRUCTURE, MORPHOLOGY, AND BIOLOGY OF IVD CELLS

**Figure 1:**
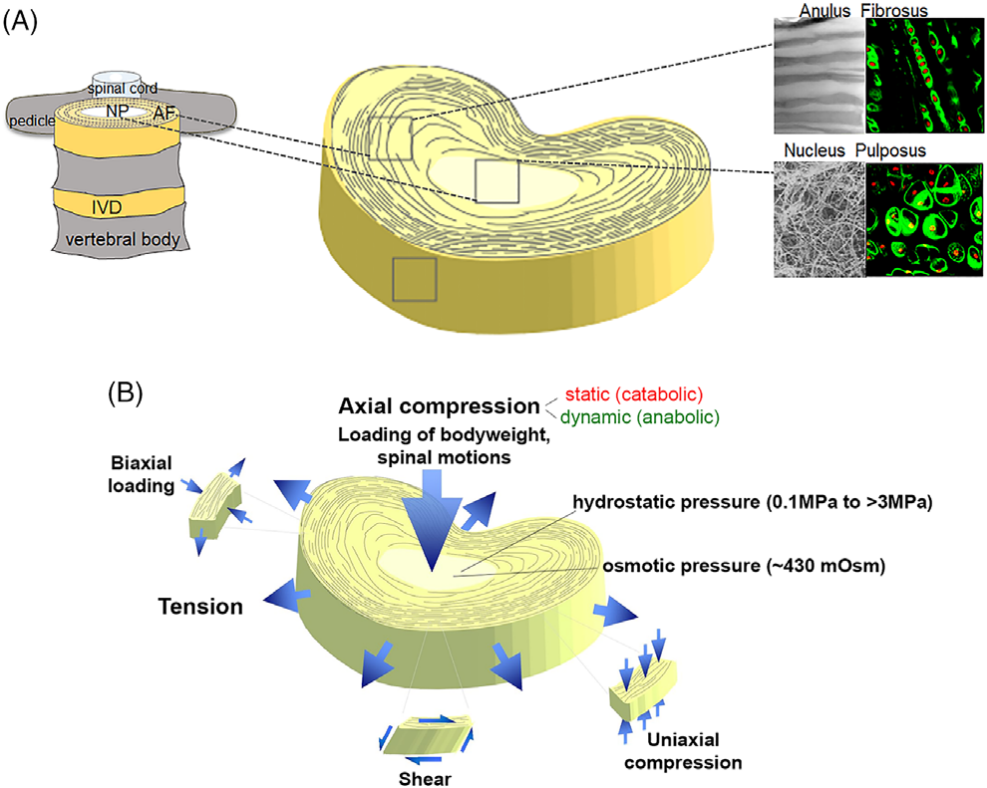
(A) Schematic representation of spinal anatomy (left) and the heterogeneous tissue composition of the intervertebral disc (IVD) (right). Insets (right) show matrix organization (scanning electron microscopy, gray) and cell organization (fluorescence) for anulus fibrosus (AF) and nucleus pulposus (NP) regions. AF matrix is composed of aligned collagen I fiber bundles, while a much random distribution of collagen II bundles and proteoglycan is observed in the NP. Fluorescence insets are rat IVD cells stained for collagen VI (green) and nucleus (red) revealing the different cellular arrangement in each tissue area. Adapted from Cao et al^174^. (B) Mechanical deformation of the IVD. Axial compression can be either catabolic or anabolic depending on mode (static, dynamic), magnitude, frequency, and duration. Loading of the hydrated NP matrix results in hydrostatic and osmotic pressures. When the disc is axially loaded, the NP region becomes compressed and the AF undergoes radial and circumferential tension to limit overall disc expansion in the transverse plane. Adapted from Setton et al^195^

The disc consists of structurally distinct, anatomical regions, which dictate the range of IVD cell phenotypic characteristics, including unique physiological and biological responses to mechanical stimuli^6^, ^7^. The innermost NP is a highly hydrated, gelatinous tissue containing large quantities of proteoglycans, collagens, and noncollagenous proteins^8^, ^9^. Due to the large amounts of the proteoglycan aggrecan, which carries a net negative and “fixed” charge through its compositionally major sulfated‐glycosaminoglycans, the NP is subject to high interstitial swelling and osmotic pressures during joint loading^10^, ^11^. The notochordal‐derived cells of the NP can be present with large vacuoles^12^, ^13^, ^14^, ^15^ and young, immature NP cells display clustered, tightly packed cells with smaller compacted nuclei, and surrounded by dense pockets of ECM (Figure 1A). The ratio of large vacuolated notochordal to small nonvacuolated cells in the NP region declines with maturity of the human IVD^15^. With age, these phenotypically unique cells are rarely seen and instead an increased presence of chondrocyte‐like or fibroblast‐like cells is observed^16^. (Notochordal cells in different animal models are further discussed in Sections 3.3 and 4.1) Indeed, there is increasing evidence demonstrating that differentiation of large vacuolated notochordal cells into mature NP cells may be mechanically regulated. Recent work has shown that periods of ex vivo organ culture for porcine^17^ and mouse^18^ IVD under hydrostatic pressure or hyperosmotic overloading conditions can promote the transition and differentiation of notochordal cells into NP cells. The differences in vacuolated notochordal cell population with age should be considered when addressing different studies, as cells from younger specimens may be a more appropriate fit for addressing early events that can lead to degeneration, while older samples may give more accurate information on the degenerate or aged cellular response to further environmental challenges.

**Figure 2:**
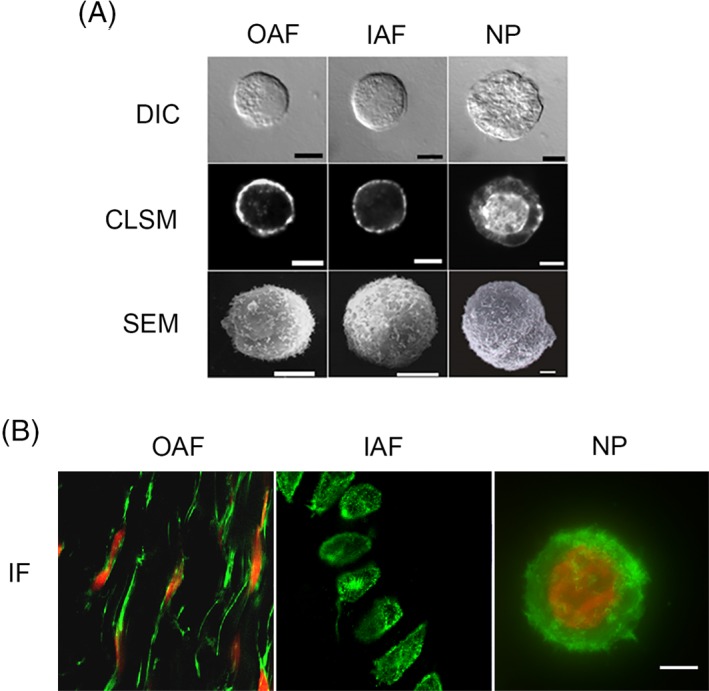
Representative images of isolated porcine cells from outer AF (OAF), inner AF (IAF), and nucleus pulposus (NP) regions. (A) Differential interference contrast (DIC, top), confocal laser scanning microscopy (CLSM, middle) depicting F‐actin (phalloidin) staining and scanning electron microscopy (SEM, bottom). Porcine NP cells are larger than OAF and IAF cells, and have a vacuolated morphology. F‐actin is localized to the cell cortex in OAF and IAF cells, but was found in abundance throughout NP cells, including the perinuclear region. SEM showed the presence of significant membrane ruffles and folds. Scale bar = 5 μm. Adapted from Guilak et al^196^. (B) Immunofluorescence of actin microfilaments (green) and the cell nucleus (red). OAF cells in situ are elongated and distributed in concentric layers mimicking the collagen arrangement. IAF cells in situ appear rounder than OAFs. Bovine NP cells (in alginate) have a dense network of punctuated cortical actin microfilaments and lack stress fibers, while OAF and IAF cells have a cortical actin distribution. Scale bar = 5 μm. Adapted from Setton et al^195^

Surrounding and constraining the NP is the AF, a lamellar and fibrocartilaginous structure containing highly organized, distinct lamellae^19^, ^20^ and largely aligned collagen fiber bundles^21^, ^22^, ^23^. Specifically, AF is constructed of concentric lamellae of alternating alignment, with each lamella composed of parallel collagen fibril bundles^24^ (Figure 1A). Sheaths of elastic fibers, formed by elastin, fibrillin, and other ECM proteins, enclose these collagen fibril bundles^25^, resembling what has been described for tendon and muscle. Elastin appears to maintain the integrity of the collagen lamellae, allowing its recoiling after deformation and to contribute to the anisotropy of the AF stiffness in response to shear^26^, ^27^. Cellular interactions with ECM of the AF and other regions can be quite complex, however, with evidence of cellular projections into lamella of the AF that can contribute to stellate‐like cells and some documented evidence of cellular “gliding” and lamellar cross bridges forming during simulated motion^28^, ^29^. Containing bundles of collagen I, the outer regions of the AF help resist tensile loads arising from physiological joint motions and swelling effects that give rise to annular bulging and deformation (Figure 1B)^30^. Cells of the AF, which are derived from the mesenchyme, display a more ellipsoidal morphology with many characteristics of fibroblasts and chondrocytes^8^, ^9^, ^31^. The inner region of the AF transitions to a more collagen II‐rich niche with increased proteoglycan content, allowing it to contribute to the adjacent NP's ability to support compressive loads. Similar to NP, cells of the inner AF (IAF) are more rounded and sparsely distributed as compared to cells of the outer AF (OAF; Figure 2). Containing bundles of collagen I, the OAF help resist tensile loads arising from physiological joint motions and swelling effects that give rise to annular bulging and deformation (Figure 1B). Cells residing in the OAF exhibit a flatter morphology, with major cell radii closely aligned with collagen fibers of the organized lamellae (Figure 2). Therefore, IAF cells appear to have greater similarity with NP over OAF, in terms of morphology and gene expression^32^. The elastic fiber network also differs between NP, IAF, and OAF. In OAF, microfibrils colocalize more with elastin fibrils when compared to IAF. In NPs, microfibrils surround the cell while elastin preferentially localizes in the interterritorial ECM. Moreover, the elastin content increases with the degree of degeneration and with age in NP, OAF, and IAF, with a higher increase in the latter^33^.

The avascular nature of the IVD largely dictates the metabolism of disc cells. The primary means by which nutrients and metabolites reach the disc is diffusion through the vertebral EP and AF, which causes relatively low oxygen tension within the disc and cell metabolism largely relies on anaerobic glycolysis^34^, ^35^. This further contributes to high concentrations of lactate, the end product of glycolysis, and low pH conditions. When oxygen tension is low, cells will rely completely on glycolysis for ATP production and energy generation^36^. Disc cells are highly adapted to such a hypoxic environment and maintain maximum cell survival when oxygen tension is below 5%^37^, ^38^. The hypoxic demands of the disc thus require high amounts of glucose for glycolysis‐dependent ATP production and cell viability becomes compromised if glucose levels fall below physiologic levels (0.2 mM)^39^, ^40^, ^41^. The resulting lactate accumulation can lead to a reduced pH, if lactate is not properly cleared, and can be detrimental to cell survival. Acidic pH levels as low as 6.1 have been predicted,^42^ that is exacerbated during degeneration because the disc has diminished diffusion abilities to remove metabolic waste products. Degeneration‐induced calcified EPs block not only nutrient supply, but also clearance of metabolites, leading to lactic acid buildup and an increasingly acidic environment^43^. Few studies have compared metabolism of NP and AF in response to loading, though AF and NP cells exhibit different metabolic pathways. NP cells have higher adenosine triphosphate content, while AF cells have higher lactate production and glucose consumption^44^. The same study also showed that dynamic loading affects energy metabolism, with the effect being greater in AF.

Pronounced changes occur with aging in the IVD, where the already low cell density decreases further, along with marked changes in the ECM composition^45^. A reduction in aggrecan within the NP results in a more dehydrated tissue with a diminished ability to properly distribute and transmit mechanical loads^46^, ^47^. This in turn leads to altered AF mechanical properties as well, due to these forces being unnaturally transferred to the adjoining AF. An important characteristic of IVD degeneration is the pathological infiltration of other cell types, namely, nerve fibers, immune cells including macrophages, lymphoid‐derived T cells, Schwann cells, endothelial cells, and fibroblasts, the latter of which may be associated with increased vasculature^4^, ^48^, ^49^, ^50^, ^51^. Along with this aberrant vasculature and subsequent blood supply, there is also a reduction in nutrient diffusion^52^, ^53^, all of which are believed to support a particularly adverse cellular environment where matrix‐degrading metalloproteinases (MMPs) and pro‐inflammatory cytokines such as interleukin(IL)‐1, IL‐8, and tumor necrosis factor (TNF)‐α, exacerbate and further the progression of degeneration^4^, ^48^, ^54^, ^55^.

## 3 MECHANICAL LOADING EFFECTS ON IVD CELLS

During loading, cells of the IVD experience compressive, tensile and shearing deformation, fluid flows, pressures, and electrokinetic effects, all of which represent important regulators of cell metabolism in the IVD (Figure 1B). Cells of the separate anatomical regions are exposed to a range of mechanical loads and the biological response to a particular stimulus may depend on tissue location, magnitude, and frequency of loading^3^, ^56^, ^57^.

### 3.1 Compression

The differential response of IVD cells to loading has been most studied for compressive stimuli. Evidence consistently suggests that there is a threshold effect observed in IVD compression studies in both NP and AF that is dependent upon magnitude, duration, and frequency. Static compressive loading has been shown to induce changes in cell biosynthesis and gene expression for collagens and proteoglycans, and protease activation, as well as cell death when applied in vivo and to explants or isolated cells^58^, ^59^, ^60^, ^61^, ^62^, ^63^, ^64^, ^65^. Static compressive loading is thought to inhibit nutrient transport and necessary gas exchange that may be essential for promoting cellular biosynthesis and maintaining cell survival, whereas dynamic compression promotes convection that increases large molecular weight nutrient uptake into the disc^42^, ^66^. Interestingly, transport of small molecules such as oxygen and glucose are predominantly governed by diffusion rather than convection that are modulated under dynamic loading^42^, ^67^, ^68^, ^69^, ^70^. In terms of gene expression, one study showed 72 hours of immobilization followed by 2 hours of dynamic loading can produce damaging effects, measured by a decrease in anabolic gene expression and an upregulation of catabolic gene expression, that were not rescued by the brief period of dynamic loading^71^. In contrast, shorter periods of static loading can lead to an anabolic response in IVD cells, shown through increases in proteoglycan and collagen synthesis in a bovine explant culture system (5‐10 kg, ~0.2‐0.4 MPa)^65^. A similar effect was observed for AF cells isolated from tissue and embedded in alginate matrix, demonstrating elevated gene expression of collagens I and II, aggrecan, decorin, and biglycan for short periods of loading^64^. As has been shown for other mechanosensitive tissues and associated cell types, long periods of matrix compression give rise to altered physical signals to IVD cells and also disrupt gas and nutrient transport that is essential for maintaining metabolism. Transport properties of human AF are dependent on the magnitude of applied compressive strain, where transport decreased with increasing deformation, likely due to fluid exudation and reduction in pore size with compression^72^, ^73^. AF diffusivity is also anisotropic, with lower diffusivity in the radial direction than in the axial or circumferential directions, indicating that nutrient transport in human AF is anisotropic. This behavior is likely a consequence of the layered structure, unique collagen architecture, elastic fibrils arrangement, and high complexity of the ECM of AF tissue^73^, ^74^.

Dynamic compressive loads change the cell‐specific expression of crucial matrix genes (eg, collagens I and II, aggrecan) and catabolic genes (MMP‐1, ‐3, ‐13, ADAMTS‐4), but the degree of response is conditional upon frequency and magnitude^71^, ^75^, ^76^. Overall, an anabolic response is more likely elicited from dynamic loading, whereas static loading tends to produce a catabolic response. In a bovine IVD organ culture study, it was found that dynamic compression (0.2‐1 MPa) increased metabolic rates and biosynthesis of ECM molecules, without any structural changes or impaired mechanical properties, indicating there is some degree of healthy loading conditions required for all regions of the IVD^77^. Lower loading rates to cells in IVD matrix and as isolated cells (<0.5 Hz) have been shown to maintain proteoglycan content^75^, ^78^ and support normal disc metabolism^79^, whereas increased loading frequencies result in higher amounts of apoptotic cells^80^. Cell death has also been noted in cells in the NP and AF when the dynamic compressive magnitude reaches levels greater than 1.3 MPa^76^, although low frequency of loading and long periods of duration (>24 hours) also modulate this effect^59^, ^60^, ^63^, ^71^, ^76^. One study used a rat tail dynamic compression model to demonstrate that discs loaded for 2 weeks at 8 hours/day expressed higher levels of anabolic and anti‐catabolic genes compared to static compression^81^. However, when discs were loaded at the same magnitude for 8 hours/day out to 8 weeks, early signs of degeneration were noted through decreased TIMP‐3 and increased ADAMTS‐4 expression, which can promote matrix degradation. Thus, dynamic compression leads to an anabolic response when applied daily, but initial signs of degeneration may begin with extended dynamic loading duration.

Compressive loading also regulates the synthesis and consumption of glucose and ATP in the IVD^42^, ^44^, ^65^, ^82^, ^83^. Both static and dynamic loading conditions have been shown to significantly increase lactate accumulation in the AF and NP regions, likely a consequence of lactate production, occurring contemporaneously with increased ATP content and decreased pH^84^. It was also noted that lactate accumulation was higher in the 1 Hz compression group compared to the 0.1 Hz compression group, suggesting frequency‐dependent differences. Isolated porcine NP and AF cells in a three‐dimensional (3D) agarose culture model that were subjected to various mechanical loads (which had previously shown the greatest changes in biosynthetic activity by mesenchymal stem cells [MSCs]) demonstrated increased glucose consumption and lactate production and were further shown to produce higher levels of ATP compared to cells exposed to decreased loading conditions^82^. Given the fact that proteoglycan synthesis is a highly ATP‐demanding process in which it participates as an energy source and as a building block, it seems likely the increased ATP found under certain conditions may interact with proteoglycan synthesis^85^, ^86^. ATP regulates various cellular activities through purinergic signaling pathways, specifically by way of ATP hydrolysis to produce ADP and adenosine, which directly modulate cellular activity through purinergic P2Y and P1 receptors^87^, ^88^, ^89^, ^90^. Isolated porcine NP and AF cells have been shown to express P2X_4_ purinergic receptors and when this receptor is inhibited, disc cells lose the ATP‐induced membrane potential response observed when stimulated with exogenous ATP^91^, ^92^. Thus, influence of mechanical loading on disc cell energy metabolism and bioactivity may affect mechanotransduction pathways.

Dynamic loading has been shown to modulate cellular responses and mechanotransduction for many cell‐laden 3D tissues such as cartilage and IVD. For the IVD, finite element modeling of dynamic compression is associated with increased oxygen concentration throughout the tissue^42^ that will be associated with elevated metabolism, confirming many of the experimental observations described. This elevated transport of oxygen to cells in the IVD is associated with increased glucose consumption and lactate production (ie, energy conversion) that may be associated with elevated proteoglycan and collagen synthesis^93^. While oxygen transport has been reported to be sensitive to loading frequency and amplitude, the effect of dynamic loading on transport is dependent on the molecular weight of the solute. In dense hydrated tissues, small molecules are less sensitive to dynamic loading and associated changes in pore size, whereas larger molecular weight solutes, such as growth factors which dramatically influence anabolism (eg, IGF‐1, TGF‐β, FGF, PDGF), undergo enhanced nutrient transport under dynamic loading that is mediated by enhanced convection^94^, ^95^, ^96^, ^97^, ^98^. Thus, many of the reported effects for dynamic compressive loading on metabolism may in fact derive from dynamic compression‐related regulation of nutrient transport and associated receptor signaling^99^.

**Figure 3:**
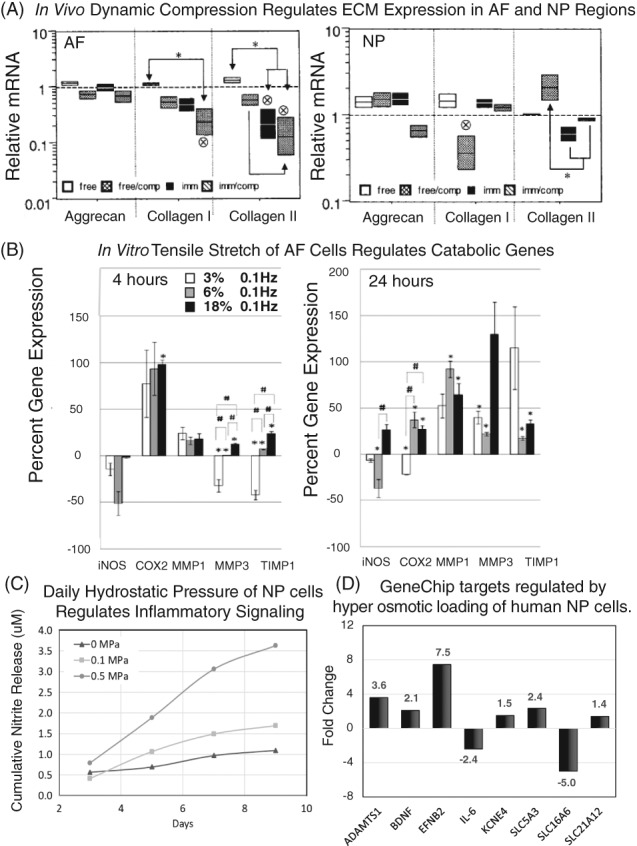
During loading, cells of the intervertebral disc (IVD) experience compressive and tensile deformation, as well as hydrostatic and osmotic pressures, among other physical effects. Mechanical signals are important regulators of cell biological responses in the IVD. (A) Dynamic compression applied to rat tail IVDs in vivo differentially regulates extracellular matrix (ECM) expression (aggrecan, collagen I, collagen II) in the anulus fibrosus (AF) and nucleus pulposus (NP) regions to the same magnitude of loading (adapted from Maclean et al^71^). (B) Applied stretch to AF cells in vitro results in a magnitude and duration dependent changes in inflammatory genes (iNOS, Cox2) and protease expression and regulation (MMP1, MMP3, TIMP1, adapted from Sowa et al^109^). (C) Daily applied hydrostatic pressure induces inflammatory signaling in NP cells in vitro in a magnitude and duration dependent manner. Adapted from Shah and Chahine 121. (D) Hyperosmotic loading of human NP cells induced decreased cell volume and cytoskeletal reorganization, as well as regulate genes include neurotrophins (BDNF), pro‐inflammatory cytokines (IL‐6), proteases (ADAMTS1), and multiple solute transporters and ion channels (adapted from Boyd et al^135^)

The distinct anatomical regions of the IVD are known to respond differently to compression, as will isolate cells from their respective tissue in terms of health and age. Many responses are similar for the IAF and NP tissues; however both short‐ and long‐term studies show that the more fibrocartilaginous OAF is not equally responsive to low to moderate loading magnitudes^61^, ^62^, ^65^. Rodent tail models have shown increased protease activation (MMP‐2) and decreased protein gene expression (collagen II and aggrecan) after 4 to 7 days of an in vivo loading protocol that was observed in the IAF and NP (Figure 3A)^58^, ^79^. The NP exhibited a frequency‐dependent response while the AF had a magnitude‐dependent response in collagen synthesis, which is believed to be due to the separate regions experiencing differences in hydrostatic and deformation‐related stimuli^79^, ^100^. The observed cell‐specific effects likely apply to duration of loading as well, where longer periods of loading (8 hours/day) can be beneficial for NP but damaging for AF^81^. Distinctions between the biological responses to load are related to the different magnitudes of deformation, fluid‐flow, and associated electrokinetic effects for cells in different tissue regions^24^, ^42^, ^44^, ^70^, ^101^. Age is also known to play a role in the response of NP and AF cells to dynamic compression, where mature cells lose the ability to maintain homeostasis to the degree that young cells do^83^. Overall, little is known of the mechanisms that govern these responses but could in part be attributed to transport of chemokines like growth factors, activation of mechanoreceptors, related ion channel activation, and cytoskeletal reorganization. These specific signaling mechanisms that contribute to the observed anabolic and catabolic mechanobiological responses of NP and AF cells have been little studied in the context of healthy, and more so in degenerated IVDs. A summary of known and proposed mechanisms regulating IVD mechanobiology is presented in Section 4.

### 3.2 Tension

Most mechanical loading conditions present tensile strains in the AF region of the IVD, though the magnitude and response of these stimuli likely varies depending on the anatomic zone. in vivo^102^, ^103^ and explant studies have previously demonstrated tension will lead to a decrease in proteoglycan and collagen synthesis in the IVD; however, this effect may be magnitude dependent with modest inhibitory effects of physiological magnitudes of tension on isolated AF cell biosynthesis^104^. The OAF region has been shown to experience biaxial stretch during functional loading, with magnitudes between 4% and 6% during flexion and extension with minimal AF biologic response^105^, ^106^. More studies have shown that isolated AF cells at low magnitudes (1%) and physiologic frequencies (1 Hz) of tensile strain maintain proteoglycan production, consistent with a modest response to strain^107^. Loading frequencies outside of this physiologic range, however, can cause a catabolic response and upon exposure to frequencies greater or less than 1 Hz, AF cells lose the ability to maintain important matrix production^108^, ^109^. Increasing strains (5%‐20%) as well can lead to a catabolic response as shown through increased MMP‐3, COX2, nitric oxide (NO), TNFα, and decreased proteoglycan production (Figure 3B)^107^, ^109^, ^110^. It has been suggested that this transition from anabolic to catabolic response occurs during periods of continuous strain and may be explained by the cells' loss of energy production necessary to maintain biologic processes.

In contrast, the NP is not believed to experience high tensile strains under physiological loading, with no major changes in the NP observed (with the exception of ADAMTS‐4, which was also confirmed in a separate study^111^). Under hyperphysiologic stretch of 10% (applied at 0.5 Hz) or 20% (applied at 0.05 Hz), NP cells respond with increasing cell proliferation and collagen synthesis^112^. Short‐term hyperphysiologic stretch (20%) of NP cells also activates innate immune receptors, such as Toll‐like receptors (TLRs), in vitro^110^. It is believed that these differential responses of NP and AF cells, likely reflect cytoskeletal adaptation to their respective in situ mechanical demands, with NP cells experiencing hydrostatic and/or compressive pressure and AF cells subjected to tensile strains.

### 3.3 Hydrostatic pressure

The NP is highly hydrated, and therefore cells within the NP experience hydrostatic pressure when loaded. Hydrostatic pressure is distinct from other loading exposures due to the presumed conservation of cell and pericellular matrix volumes. Hydrostatic pressure magnitude also varies diurnally in a manner that depends on spinal alignment and physical activity^113^, ^114^. Both experimental and computational methods have been used to estimate the magnitude of hydrostatic pressure that disc cells are exposed to during activity. Hydrostatic pressure magnitudes range from 0.1to over 3 MPa, with baseline magnitude of around 0.1 MPa applied at all times regardless of posture or activity^115^, ^116^. With aging, hydrostatic pressure magnitude is likely reduced due to lower proteoglycan and water content of the disc^17^, ^117^. Exposure of disc cells to hydrostatic pressure is known to influence disc cell biologic responses in duration, magnitude, and frequency‐dependent manner, with some species‐specific variations observed. In general, higher magnitudes of hydrostatic pressure (>3 MPa) can lead to degenerative responses in IVD cells mediated by an increase in MMPs and reduction in ECM expression and synthesis when applied to NP cells seeded within a 3D culture system or within tissue explants^113^, ^118^. Hydrostatic pressure overloading of 3 to 10 MPa applied at frequency greater than 1 Hz on cells from bovine or porcine NP^119^, ^120^, ^121^, porcine AF^122^, as well as human IVD cells^120^ appear to decrease anabolic gene expression and increase catabolic gene expression. Whereas hydrostatic pressure (>3 MPa) applied at 20 Hz to rabbit IVD cells showed a higher ratio of collagen synthesis to degradation^123^. When disc cells are maintained under a physiological pressure range (0.3‐1 MPa), increased matrix synthesis occurs^56^, ^124^, ^125^, ^126^, ^127^. However, within this range, differing responses have been observed. Some studies have shown an upregulation in ECM proteoglycan synthesis, while others show a downregulation in ECM and upregulation of catabolic signaling (Figure 3C)^124^, ^125^, ^126^, ^127^, ^128^. Similarly, while some studies find that collagen synthesis to be upregulated in this range of loading magnitudes, others show decreased expression of collagens I and II at the gene level^120^, ^121^, ^124^, ^127^, ^128^. Some of these changes may be attributed to volumetric or cell morphological changes. Moreover, hydrostatic pressure effects appear to be dependent on cell density^121^ of tested samples, with higher cell density leading to higher cell stress under hydrostatic pressure.

Species‐specific variations may be due to phenotypical and morphological differences in NP cells which are derived from the notochord. In some species (eg, rodents and porcine), the NP cells maintain a notochordal morphology into adulthood, while others (human and bovine discs) have small nonvacuolated single NP cells surrounded by a dense ECM^129^. While the factors that drive this cellular transformation or transition are yet to be fully understood, hydrostatic pressurization may be a regulator of such transformation. One study found that dynamic pressurization can induce a transition of notochordal cells to a mature single cell phenotype in a porcine explant model^17^; however, hydrostatic pressure did not alter the expression of certain NP phenotypic markers (cytokeratin 18 or brachyury) in vacuolated notochordal NP cells. On the other hand, hydrostatic pressure was found to be a modulator of NP phenotypic markers (cytokeratin 19 and N‐cadherin) in nonvacuolated NP cells^121^. The presence of vacuoles has been postulated to function as a regulator of cell volume and tonicity during rapid osmotic stress, protecting the cells from potentially damaging swelling pressures^130^. Therefore, species variation is likely occurring due to differences in biomechanical function and biological uniqueness. Results from hydrostatic pressure studies continue to challenge our understanding of mechanisms that contribute or regulate IVD responses, and more work is needed to verify specific cell‐ and loading‐dependent responses.

### 3.4 Osmotic pressure

The osmotic environment of the IVD, resulting from a combination of mechanical deformations and fluid redistribution, can vary locally depending on biochemical composition and local volumetric change^7^. Osmotically induced swelling due to hypotonic conditions draws water into the disc tissue to maintain hydration, and causes volumetric gain^50^. Urban^7^ has demonstrated that the interstitial osmotic pressure is considerably higher in the disc (~430 mOsm) compared to plasma and the changes that occur on IVD loading following compression are closely associated with changes in the density of proteoglycans and extracellular hydration. An important component in maintaining and modulating osmotic forces, the presence of large, aggregating proteoglycans in the NP, particularly aggrecan, creates a high negative fixed charge density that allows tissue swelling and mechanically driven fluid flow with associated electrokinetic effects^11^, ^101^, ^131^, ^132^. This contributes to the primary biomechanical function of the NP where osmotic pressurization of the NP serves to balance loads endured by the spine through its distinct osmotic and water‐binding properties 50. Proteoglycan production is greatest under physiologic osmolality (~430 mOsm), but decreases if higher or lower osmotic pressures are reached^7^, ^133^, ^134^. Osmotic stimuli may exert its effects at the transcriptional level as altered (hypo‐ or hyper‐) osmolality has been shown to broadly influence mRNA expression of genes related to cytoskeletal remodeling, osmolyte transport, pro‐inflammatory cytokines, and neurotrophins (Figure 3D)^135^. Although it is known that the response to altered osmolality is regulated at the transcriptional level, exact mechanisms by which osmotic pressure modulates IVD cell metabolism are not known. Increased osmolality can also decrease DNA synthesis or even induce DNA damage, which has been shown to push human IVD cells into low or ceased proliferative states where cell cycles can be delayed at the G2/M and G0/1 phases^136^. This hypertonicity‐induced cell cycle arrest is partly mediated by MAPK/p53 activation and reduced ERK/Akt stimulation^136^, ^137^, ^138^.

Due to alterations in proteoglycan and hydration content in the aging and degenerate matrix, these osmotic pressure changes in the IVD are some of the most drastic changes known. Distinguished by its hypoxic, avascular environment with limited nutrient supply, the normal healthy disc maintains a relatively high osmotic pressure of the proteoglycan‐rich ECM^7^. These characteristics are markedly reduced, likely initiated by a loss in proteoglycans that can lead to decreased osmotic pressure and impaired water binding ability of the disc ECM. One study investigated the effect of long‐term osmotic loading on structural and functional properties of juvenile and adult bovine NP and EP tissues found that culture under osmotic conditions that recapitulate the healthy disc environment in situ (400 mOsm/kg) promoted matrix production and mechanical function of engineered tissues^139^. The persistence of hypoosmotic conditions in degenerate discs may not only allow for increased IVD cell proliferation^137^, ^138^, but may also accelerate senescence^137^, ^140^.

## 4 SUBCELLULAR MECHANOTRANSDUCTION

### 4.1 Cytoskeleton of the IVD

The response of cells to loading, and the specific mechanotransduction pathways mediating responses to load, is dependent on cell morphology, cell‐cell interactions, and cell‐ECM interactions. Loading of cells can act directly on cytoskeletal networks by promoting F‐actin reorganization, which can act as a mechanism for transducing mechanical stimuli to the nucleus^64^, ^106^. In healthy, rounded NP cells, F‐actin is distributed cortically around periphery of the cell and is distributed as short punctate fibers throughout the cytoplasm (Figure 2). NP cells in vivo and in situ lack stereotypical F‐actin stress fibers^13^, ^141^. In OAF cells, F‐actin is distributed throughout the cytoplasm extending into the cell processes, reflecting a fibroblast‐like morphology. Actin expression levels are higher in OAF compared to NP^141^. F‐Actin is the major cytoskeletal element contributing to stiffness of NP and AF cells^142^, with actin integrity playing a key contribution to cellular viscoelastic properties.

NP and AF cells also rely heavily on the presence of additional cytoskeletal components, such as intermediate filaments (IFs) and tubulin, the latter of which forms an extensive meshwork throughout the cytoplasm of NP and OAF cells, where expression level is higher in NP than OAF cells, but no appreciable differences in organization by zone have been observed^141^. IF proteins are classified into five main groups, with the following present in cells from the IVD: keratin 18 and 19 (Type I), keratin 8 (Type II), vimentin (Type III), and nuclear lamins (Type V). These specific forms of IF proteins have come to be important NP and AF cell‐specific molecular markers^143^. IFs can withstand large deformations and at the same time they can be highly sensitive to small forces in the context of cell signaling^144^. Vimentin IF spans the cytoplasm from the plasma to the nuclear membrane in NP cells, whereas in OAF cells, vimentin filaments also extend into the cellular processes. Interestingly, AF cells only express vimentin IF, whereas NP cells coexpress cytokeratins and vimentin. This broad IF coexpression in NP cells is shared by cells of other tissues exposed to fluid or semifluid environments, and with high concentrations of hyaluronic acid (ie, ovaries, endometrium, kidney, epithelial cells of the thyroid tissue, Sertoli cells, endoderm of embryo and notochord)^145^. The expression of cytokeratin is higher in NP compared to AF and chondrocytes^145^. While keratin IFs are an essential component of cells that experience shear loading^146^, vimentin has been more associated with protection against compressive forces (eg, fibrocartilage cells) and during development^147^. Vimentin IFs contribute little to cortical stiffness, but they appear to regulate intracellular dynamics by stabilizing organelles in the cell^148^.

During development as the notochord narrows to form the spine, compressive forces are exerted on the IAF while tensile forces are exerted on the OAF^149^. Correspondingly, AF cells induce formation of actin stress fibers and trigger the deposition of a fibrous ECM^149^. During this process, cells form stress fibers that mirror the alignment of fibronectin and collagen bundles indicating that internal cellular structure drives ECM organization^149^. While actin is essential for this process, vimentin seems more related to a later differentiation stage of AF cells when levels of compressive forces increase. After birth, there is reorganization of AF cytoskeletal elements and cells seem to rely more on the already deposited ECM^149^. In the NP, actin distribution changes with development; however, the changes depend on the presence of a vacuolated (notochordal) compared to nonvacuolated NP cells in a species. Notochordal NP cells form interconnected clusters of large vacuoles that have dense cortical actin with an actin filled cytoplasm^12^, ^13^, ^14^, ^15^. With postnatal development, vacuolated NP cells and their cytoskeleton persist in most species (mouse, rat, rabbit, and pig). In a few species (sheep, mongrel dogs, and beagles), vacuolated NP morphology and cytoskeleton change to a nonvacuolated type. Nonvacuolated NP cells appear unconnected and have robust but distributed staining for F‐actin, vimentin, and cytokeratins. In nonnotochordal species, no major differences have been observed in actin, tubulin, or vimentin organization with skeletal maturity^141^. Actin and tubulin expression do not appreciably change with age; however, vimentin expression decreases with age in OAF, suggestive of a transcriptional shutdown in a bovine model^141^. In human NP cells, the expression of cytokeratins and vimentin decrease with development and postnatal maturity^145^. Normal mature human discs have more heterogeneity in cellular cytoskeleton than is observed in animal models. For example, stellate cells with multiple cellular processes that are positive for F‐actin and vimentin have been observed in the IAF and NP of both normal and pathological human discs, which may reflect a response to abnormal loads or microenvironments.

**Figure 4:**
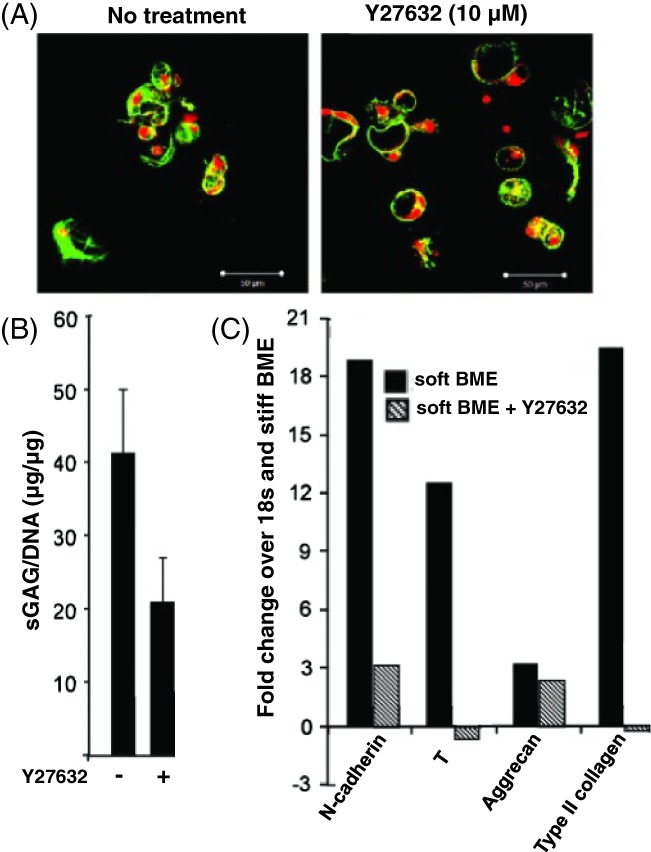
(A) When porcine nucleus pulposus (NP) cells are treated with the ROCK inhibitor Y27632, multicell cluster morphology is lost, and single cells are observed with little cell–cell interactions. (B) ROCK inhibition also promotes decreased biosynthetic s‐GAG activity and (C) expression of the NP markers n‐cadherin, T (brachyury), aggrecan, and collagen II (adapted from Hwang et al^151^)

Actin cytoskeletal remodeling is largely mediated by the RhoA/ROCK signaling pathway, which is involved in many responses such as cell migration, polarization, stress fiber, and adhesion formation^150^. RhoA/ROCK signaling is also altered in response to mechanical stimuli, which creates complex interactions between morphological and biomechanical changes. Porcine NP cells normally form cell‐cell interactions and cell clusters; however, this function is lost when cells are treated with ROCK inhibitors (Figure 4A), underscoring the role of ROCK‐dependent RhoA GTPase activation in regulating cell clustering in young NP cells^151^. Under these conditions, treated cells also have decreased ECM synthesis and expression of NP markers brachyury, and N‐cadherin (Figure 4B,C), suggesting that ROCK pathway modulates cell phenotype and morphology. These observed coincident changes reported for cytoskeletal organization and cell biosynthesis in IVD cell mechanobiology may be related to the ROCK signaling pathway.

### 4.2 Cytoskeletal responses in mechanical loading of IVD cells

Studies of isolated IVD cell to specific physical stimuli reveal broad changes in cell morphology and suggest that cytoskeletal reorganization as one mechanism by which IVD cells respond to physical stimuli. For example, cell volume changes occurring in response to hypoosmotic or hyperosmotic stress can occur only with associated F‐actin remodeling to accommodate the change in cell volume. Early studies showed that NP cell response to hypoosmotic stress is much slower than that for the AF cell, due to the dense cytoskeleton of the juvenile NP cells^142^, ^152^. Cellular differences in cytoskeleton appear to be the principal difference regulating differential responses of AF and NP cells to osmotic loading with associated effects on other signaling mechanisms (eg, calcium signaling). As NP or AF cells undergo changes in conformation during osmotic swelling, there may be an associated activation of mechanosensitive channels, allowing calcium and associated cellular signaling to mediate cell growth, differentiation, and motility^134^, ^142^, ^153^. These intracellular calcium transients were shown to be different for NP and AF cells and are, in part, modulated by the actin cytoskeleton's stability. Indeed, NP cells appear to be more sensitive to actin disruption as demonstrated by increased calcium spikes/responses under osmotic stress compared to AF cells^142^. Interestingly, a limited number of studies have investigated ion channels, including mechanosensitive channels, and their role in IVD mechanotransduction. One study found that osmolarity changes associated with proteoglycan degradation in IVD organ cultures can alter TRPV4, an osmosensing calcium channel^154^. A proteomic analysis^155^ identified candidate ion channels that are consistently and highly expressed in IVD tissue (Table 1), with zonal differences in NP and AF. Despite a current lack of knowledge of mechanosensitive ion channels in IVD cells, they are likely an important regulator given the presence of osmotic stimuli and membrane stretch in cell volume regulation, which may activate mechanosensitive ion channels.

**Table 1:** Gene array data of ion channels found to be differentially expressed in AF or NP regions of the IVD (results from a rat Affymetrix data set, adapted from Tang et al^155^)

Higher in AF than NP	SCN1A	Sodium voltage‐gated channel alpha subunit 1
	KCNK5	Potassium channel subfamily K member 5
	KCNC3	Potassium voltage‐gated channel subfamily C member 3
	TRPV1	Transient receptor potential cation channel subfamily V member 1
	KCNMA1	Potassium calcium‐activated channel subfamily M alpha 1
Higher in NP than AF	CACNA1B	Calcium voltage‐gated channel subunit Alpha1 B
	CACNA2D1	Calcium voltage‐gated channel auxiliary subunit alpha2delta 1

Abbreviations: AF, anulus fibrosus; IVD, intervertebral disc; NP, nucleus pulposus.

AF cells isolated from the ECM undergo cell morphology changes and exhibit a tremendous capacity to reorganize their cytoskeleton in response to compression or tension. Studies with immature bovine IVDs showed that AF cells respond to applied cyclic tensile strain (10%, 1 Hz) with increases in the expression of β‐actin and β‐tubulin at both the transcriptional and protein level, while vimentin expression was decreased^106^. Together with this, actin in the AF cells formed a large number of stress fibers in response to tensile stretch accompanied by a marked cell elongation; in contrast, microtubules and vimentin filaments were less affected^106^. It was also observed that widespread remodeling of cytoskeletal components is primarily restricted to OAF cells in response to cyclic tensile strain, with increased F‐actin turnover and increased β‐actin, β‐tubulin, and collagen I expression. Supporting these findings, experiments show that human AF cells reorient themselves perpendicular to the direction of applied uniaxial stretch in a fashion dependent on actin and focal adhesions, and undergo apoptosis if this reorientation is restricted using micropatterned membranes that define cell orientation under loading^156^.

Under compressive loading, changes in cytoskeletal responses have been observed that vary with the zonal origin of the IVD cells. AF cells subjected to unconfined compression (25% strain) responded to mechanical deformation, with increasing ECM expression, increased vimentin expression and increased vimentin polymerization. In contrast, NP cells were not responsive to the same mechanical loading conditions. These distinctions are likely attributed to differences in strain magnitudes experienced in situ, but may also be due to the presence of a more diffuse and stiff cytoskeleton in the notochordal NP cells, which may restrict deformations or cell shape changes with loading.^64^

### 4.3 Cell‐matrix interactions

Integrins are transmembrane glycosylated proteins that mediate interactions of the cell surface with the extracellular environment. Structurally, integrins are heterodimers comprising two noncovalently associated subunits, referred to as the α and β subunits, which combine to form 24 distinct integrin receptors. Integrins are grouped into subfamilies based on ligand interactions. NP cells express integrin subunits α_1_, α_2_, α_3,_ α_5_, α_6_, α_v_, β_1_, β_3_, β_5_, β_6_, and β_8_ 57, 157, 158, 159, 160, while AF cells express α_1_, α_5_, α_v_, β_1_, β_3_, β_5_, and β_6_ integrin subunits^160^. In the context of the disc, the family of RGD integrins and laminin binding integrins has been best studied. RGD (arginine‐glycine‐aspartic acid) is a ligand motif that certain integrins recognize, including the fibronectin receptor α_5_β_1,_ which is present in the ECM. Integrins also mediate interactions with other important constituents of the ECM, including multiple isoforms of collagens, laminins, and small glycoproteins^158^, ^159^, ^161^, ^162^, ^163^. Integrins acts as force sensors and are essential in cytoskeletal mechanotransduction with focal adhesions.

**Figure 5:**
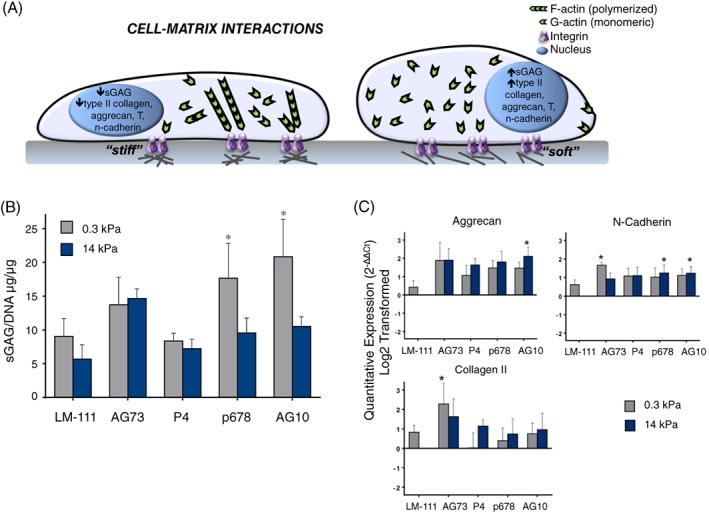
(A) Cell‐matrix interactions can regulate disc cell phenotype and bioactivity through stiffness‐ and ligand‐mediated effects, where rounded cells observed on “soft” matrices have increased production of nucleus pulposus (NP) markers and sGAG but flattened cells on “stiff” substrates can have decreased sGAG production. (B) Ligand presentation can overcome some stiffness‐mediated effects, as shown by Bridgen et al^161^ with primary human NP cells that only exhibit increased biosynthetic activity on soft substrates with select laminin‐mimetic peptides. NP cells were cultured on surfaces conjugated with LM‐111 (full length laminin), AG73 (syndecan peptide), P4 or P678 (α3 integrin receptor peptides), or AG10 (α2, α5, α6, β1 integrin‐recognizing peptide). (C) These select ligands can also produce differential NP marker expression independent of substrate stiffness

In evaluation of regional variations of integrins, the expression of α_5_β_1_ was particularly prominent in the cells from the human NP and IAF, with significantly lower numbers of immunopositive cells in the OAF^57^. A recognizable difference between NP and AF ECM was the finding of the uniquely expressed laminin isoforms in NP tissue, LM‐111, LM‐511, and LM‐332, compared to AF^163^. Immature NP cells also show expression of integrins that can mediate interactions with these laminins (α6β4, α3β1, and α6β1)^163^ to promote cell attachment, as well as upregulated proteoglycan synthesis and elevated mRNA for a subset of NP ECM molecules. It has further been shown that certain laminin‐mimetic peptides induce higher levels of biosynthetic activity compared to others (Figure 5B) and can even express statistically higher amounts of NP markers despite a stiff substrate presentation (Figure 5C). The staining of laminin seems to decrease with age in several species^164^ and their appearance on young individuals may be related to its role as component of basement membrane that surrounds the notochord during its formation and differentiation^165^.

Many studies have revealed that cell interactions with matrix proteins through integrin binding contribute to activation of ERK and MAPK pathways that may amplify or attenuate signaling through other transduction pathways such as growth factor binding^166^. Indeed, ERK phosphorylation can be confirmed for NP cell binding to laminin in vitro but has not been specifically linked to a downstream biosynthetic event through ERK inhibition^117^. Additional studies are required to verify the precise role of ligand interactions with integrins on NP and AF cells, and to confirm a role for ligand density (eg, collagen or laminin density, RGD or other peptide density)^161^ and ligand specificity in activating cell signaling through ERK, MAPK, or other pathways.

An additional role for integrins is associated with the release and activation of latent TGF‐β that is stored in the ECM, bound to a peptide^167^. Some integrin subunits have been shown to participate as docking points to allow proteolytic cleavage of immobilized TGF‐β from the docking peptide, and to enable generation of cell traction that will mechanically separate TGF‐β to enable its activation^167^. TGF‐β is critical for the disc development at embryonic stage and for its postnatal maintenance^168^, ^169^ and regulates connective tissue growth factor CCN2, implicated in synthesis of aggrecan^170^. Both excess and deficiency of TGF‐β activation can be detrimental for IVD^157^, ^169^, ^171^, ^172^. In this manner, TGF‐β‐receptor‐mediated signaling could be regulated by integrin engagement with the ECM; however, investigations on changes in TGF‐β activation with disc aging and changes in ECM stiffness may provide further insight into disease related changes.

### 4.4 Cell‐cell interactions and the cadherin‐catenin pathway

Cells of the NP and AF interact with neighboring cells in a manner that varies with age, and that is distinctly different between these two regions. Some cells of the AF form elongated cell “arrays” or may spread into flattened cells as in the case of the stellate interlamellar AF cell. These cells may interact with adjacent cells through E‐cadherin mediated junctions that are known to be key regulators of cell signaling in multiple cell types. AF expression of E‐cadherin appears to be independent of age and matrix stiffness^173^, and little is known about the relationship between E‐cadherin expression and cellular responses to physical stimuli.

**Figure 6:**
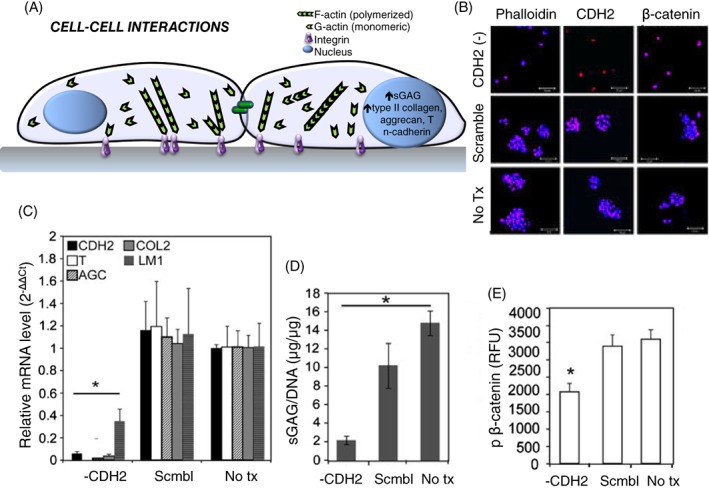
(A) Effects of cell‐cell interactions of the intervertebral disc have been observed in primary human nucleus pulposus (NP) cells where promotion of cell‐cell interactions via soft substrates or N‐cadherin (CDH2) signaling leads to enhanced biosynthesis and NP marker expression. (B) Hwang et al 117 demonstrated that the ability to form rounded multi‐cell clusters is lost when CDH2 is depleted. (C) When NP cells lose the ability to form cell‐cell interactions in the absence of CDH2, there is a significant loss of expression of NP markers and (D) decreased biosynthesis, (E) which is believed to be mediated by loss of β‐catenin

In contrast, cells of the juvenile NP cell are rounded and may exist in close proximity to other cells in rounded “cell‐matrix units.”^174^ These cells may interact through N‐cadherin‐mediated interactions that are shown to be essential for promoting mRNA expression of key markers of the NP phenotype, including brachyury, aggrecan, and laminin isoforms (Figure 6B‐D)^117^. These cadherin‐mediated interactions appear to promote β‐catenin phosphorylation (Figure 6E) and decreased nuclear lamin expression in human NP cells, which may be key signaling events responsible for the downstream upregulation in biosynthesis. The loss of N‐cadherin with aging could be related to a decrease in cell density with age^173^ and may affect both dedifferentiation of the NP cell phenotype and its subsequent capacity for injury‐induced regeneration or repair.

An important observation is that cells of the IVD may interact with adjacent cells while adopting rounded morphologies, as for NP cells or AF cells when isolated in vitro, or while adopting spindle and spread morphologies as for AF cells (or NP cells upon stiff substrates). Given the adaptive nature of the cytoskeleton and its important role in activating ion channels and RhoA kinases, we find that cell signaling through morphological confirmation is inextricably linked to cell signaling through cell‐cell interactions such as β‐catenin signaling. More work will be needed to isolate factors of cell morphology as distinct from N‐ or E‐cadherin mediated signaling in order to isolate the specific signaling events and their relationship to IVD cell phenotype.

### 4.5 Substrate stiffness effects

**Figure 7:**
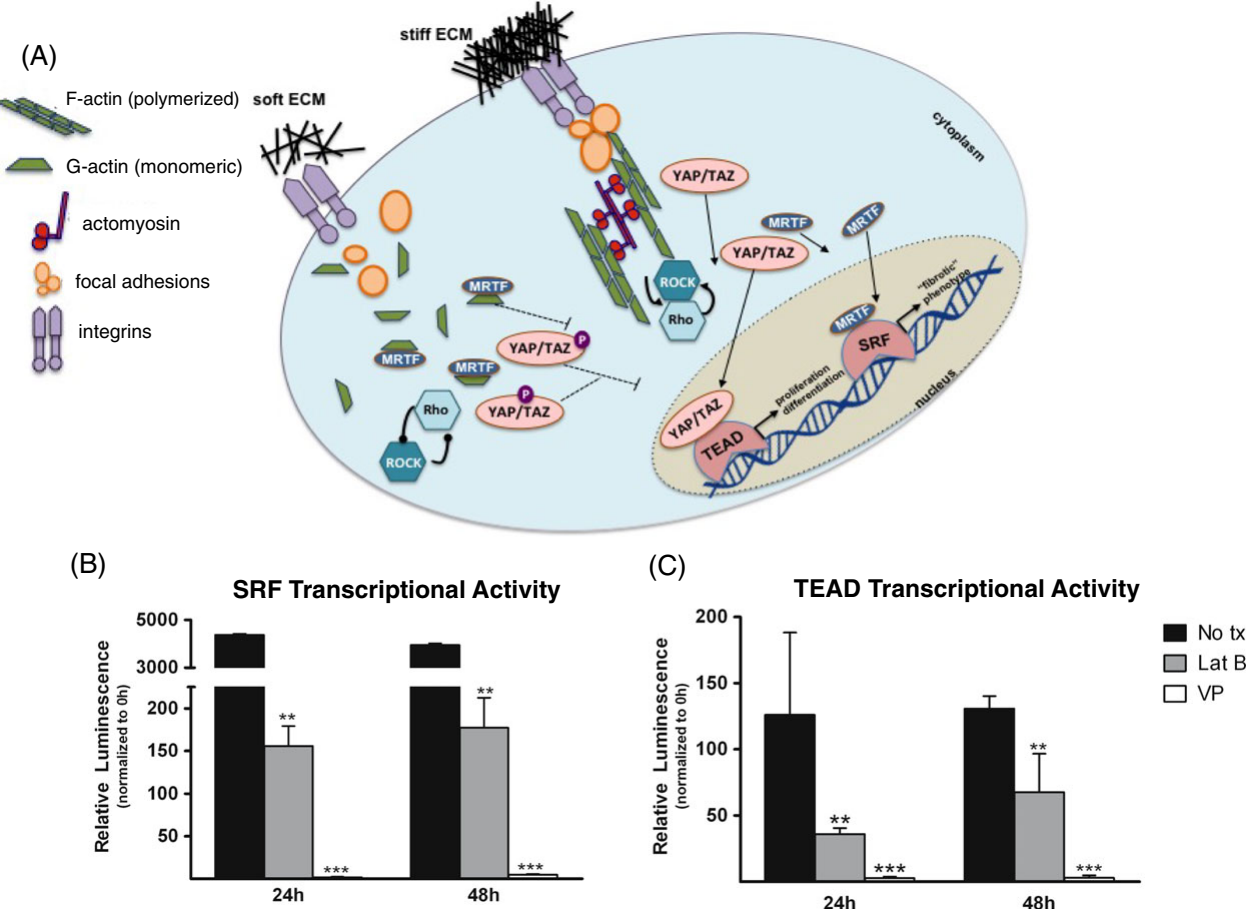
(A) Work in diverse fields has identified the critical role of actin as a direct regulator of transcription factors. SRF and TEAD are known to be active and present in primary nucleus pulposus (NP) and anulus fibrosus (AF) cells, where they are believed to be regulated by F‐actin turnover and Rho/ROCK signaling. On stiff substrates, increased polymerized F‐actin form contractile complexes with focal adhesions and actomyosin to sustain Rho/ROCK signaling to allow nuclear translocation of coactivator YAP/TAZ to bind TEAD and drive transcription of proliferation‐ and differentiation‐related genes. Similarly, coactivator MRTF is able to localize to the nucleus due to the decreased presence of monomeric actin (G‐actin) where it will bind and activate SRF to transcribe fibrotic response‐related genes. Soft substrates allow for liberation of G‐actin which sequesters MRTF in the cytosol and decreased Rho/ROCK signaling, due to the loss of the contractile actomyosin/F‐actin complex, prevents YAP/TAZ nuclear localization, thus keeping both SRF and TEAD transcriptionally silent. (B) Verteporfin (VP), a known inhibitor of YAP/TAZ, and Latrunculin B (Lat. B), which disrupts polymerized F‐actin, were cultured with SRF transcriptional reporter primary human NP cells, which resulted in significantly decreased transactivation of SRF with both Lat. B and VP treatment. (C) When TEAD transcriptional reporter NP cells were treated with Lat. B and VP, a similar decrease in transcriptional activity was observed, suggesting inhibition of a contractile cytoskeleton can prevent expression of fibrotic and aberrant cell cycle genes

We and others have found an effect of substrate stiffness on mechanobiology in IVD cells, as shown through mRNA expression and ECM biosynthesis^117^, ^151^, ^158^, ^161^, ^162^, ^173^, ^175^, ^176^, but the degree of response seems to depend largely on cell type. This is also true for chondrogenesis in MSCs, where changing substrate stiffness can impact chondrogenic events. It has been shown that lower stiffness hydrogels promote increased aggrecan, collagen, and sGAG synthesis in MSCs, indicating enhanced chondrogenic ability, while nuclear stiffening induced by stiff substrates sensitizes the transcriptional responses in MSCs as a priming effect for mechanical perturbations^177^, ^178^. The mechanism by which substrate stiffness information is transduced in IVD cells is not completely understood, but it likely relates to cytoskeletal organization and related transcriptional activators such as YAP/TAZ and SRF (Figure 7A)^179^, ^180^, ^181^, ^182^, ^183^. The cytoskeletal regulation of primary human NP and AF cells has been observed through YAP/TAZ and MRTF signaling, cofactors of known mechanically regulated transcription factors TEAD and SRF, respectively (Fearing et al, unpublished data). When isolated cells are cultured upon stiff substrates (29 kPa), transactivation of TEAD and SRF occur at significantly higher levels compared to soft substrates (0.3 kPa), where they remain predominantly quiescent and their respective cofactors stay sequestered in the cytosol. These cells cultured on stiff substrates with diminished TEAD and SRF signaling also express significantly lower amounts of aggrecan, GLUT‐1, and collagen II (Figure 7B,C) and are less biosynthetically active. Additional work has shown when F‐actin is disrupted with the small molecule inhibitor Latrunculin B, both SRF and TEAD exhibit decreased activation along with higher expression of NP markers, even when cells are cultured on stiff substrates. The presence and activity of these cytoskeletal‐regulated transcription factors in primary human disc cells indicates IVD cells rely on mechanically regulated signaling pathways.

## 5 RESPONSE TO ABNORMAL MECHANOBIOLOGY AND DEGENERATION

### 5.1 Degenerative microenvironment

Physical signals in the presence of chemical stimuli have the potential to alter cytoskeletal and biomechanical response to loading. Inflammatory stimulation of NP cells has been shown to disrupt F‐actin structure and related cell volume regulation under osmotic stress. Bovine NP cells stimulated with TNFα, a pro‐inflammatory cytokine implicated in disc degeneration, show a loss of intracellular F‐actin that is redistributed to the cell cortex^184^. Consequently, TNFα‐treated cells have an increased baseline volume and an increased hydraulic permeability^184^. Interestingly, these morphological and cell biophysical properties appear to be irreversible in vitro, even when inflammatory stimulus is returned to normal culture conditions^184^. The downstream signaling responses and associated catabolic upregulation in inflammatory environments are thought to mimic the degenerative milieu of the IVD. Evidence suggests that the response of cells from degenerate tissue to applied loading differs from cells isolated from normal IVD tissue. AF cells from degenerative tissues demonstrated a more pro‐inflammatory response to applied stretch (6% elongation, 0.1 Hz) compared to cells from nondegenerate discs, suggesting that degenerate AF cells are more reactive to exogenous stimuli. Moreover, the short‐lived beneficial effects of mechanical stretch seen in normal AF cells do not appear when degenerate AF cells are similarly stretched. Indeed, increasing evidence suggests that interactive effects of tensile stretch and inflammatory signaling in AF cells^109^, ^185^, ^186^. This may be mediated by altered cytoskeletal mechanotransduction. In AF cells, TNFα treatment increased F‐actin stress fibers α‐tubulin, which potentially sensitized AF cells to mechanical strain^185^. Short‐term hyperphysiological stretch of disc cells also activates innate immune receptors, such as TLRs, in vitro^110^. Activation of TLRs by the canonical agonist, LPS, induces cytoskeletal remodeling of NP cells, and altered biomechanical properties of NP cells, similar to the altered mechanotransduction observed in the presence of TNFα activation^184^. All these studies point to a positive feedback of degenerative microenvironments, mediated primarily by pro‐inflammatory cytokines, which alter cytoskeletal remodeling and related mechanotransduction.

### 5.2 Cell‐ECM interactions

Although one study found that physiological level of dynamic compression induced integrin α5β1 expression in rat disc explants^187^, other studies find that degenerative static compressive loading in vivo upregulate β1 expression in NP cells, and activates downstream signaling^188^. Moreover, disruption of β1 integrin signaling may underlie disc cell apoptosis induced by mechanical stress^189^ in disc degeneration models. However, in human samples, cells derived from both degenerated and nondegenerated IVDs have similar expression of integrins subunits, despite major changes in ECM content and levels with degeneration^57^, ^158^, ^159^, ^160^. No significant differences were observed in the number of immunopositive cells for the α5, β1, or α5β1 heterodimer identified in the NP or IAF of nondegenerated discs as compared with IVDs with moderate or severe histologic degeneration^57^. However, the response of cells to integrin‐mediated mechanotransduction is altered in degeneration. For example, when dynamic compressive load (sinusoidal 0.45‐0.95 MPa, 1 Hz) was applied to human NP cells, both cells from degenerated and nondegenerated sources exhibited similar reduction in gene expression of aggrecan. However, blocking of integrin binding with an RGD peptide prevented this response only in nondegenerated cells^57^. This suggests that mechanotransduction signaling is altered in degenerated tissue, in that integrins may not be directing response of degenerate cells to loading or that there are changes in the mechanosensitivity of integrins to mechanical loading in degenerate cells^57^. Studies of human AF cells derived from degenerated and nondegenerated tissue and exposed to 10% strain, 1 Hz for 20 minutes also respond differently. Specifically, there was a decrease in expression of ADAMTS4 for nondegenerated cells and a decrease in expression of collagen I for degenerated cells. Blocking integrin binding transiently prevented a response to loading in nondegenerated cells that was mediated by focal adhesion kinase; however, it failed to affect the mechanotransduction in degenerated AF cells. These results suggest that integrins participate in mechanotransduction of AF cells from nondegenerated tissue, and that alternative mechanotransduction pathways mediate responses of degenerate cells^190^.

### 5.3 ER stress

Cells also protect themselves from stresses through cellular stress responses. In one study, Chooi and Chan^191^ found differential gene expression patterns based on compression loading regimens that suggest an increase in ER stress pathway genes as a form of cell survival. In a 3D collagen model with bovine NP cells, compressive loading was applied for various durations and assessed the heat shock protein response (HSR) and the unfolded protein response (UPR). Although an increase in both UPR and HSR stress response genes were observed in the highest group of static compression, the authors note that upon load removal, HSR genes remain upregulated while UPR genes become downregulated, which may suggest HSR genes' continual expression is a protective mechanism to allow cells to recover and avoid apoptosis. This was further confirmed by showing HSR gene‐expressing cells do not become apoptotic and those that did undergo apoptosis did not express these genes postloading. This points to a master regulator of cell survival and biosynthesis following periods of static compressive loading. Similarly, hyperphysiologic cyclic stretch has been shown to increase the production of ER stress markers CHOP, GRP78, and caspase‐12 in AF cells^106^, ^192^, ^193^. In one study, the addition of caspase inhibitors was able to partially suppress the stretch‐induced apoptosis as well as NO overproduction^192^. It has been suggested that cyclic stretch‐induced apoptosis may occur through mitochondrial pathways, as cyclic stretch has been noted to cause reduction of the mitochondrial membrane potential, though other cell death pathways are likely involved.

## 6 CONCLUSION

In conclusion, cells of the NP and AF have been observed to respond to compression, tension, osmotic stimuli, and hydrostatic pressure with changes in ECM, and regulators of ECM synthesis, in a manner that differs between these two cell types. Cellular studies reveal a role for differential regulation of cytoskeletal remodeling between AF and NP cells in contributing to these differential responses, with the remodeling being specific to actin, vimentin or IF reorganization and/or expression specific to the stimuli. Cytoskeletal reorganization can activate downstream effects not only through ROCK signaling and coactivators such as YAP/TAZ, but also through volumetric regulation that may affect mechanosensitive ion channels as observed through calcium signaling. Whether the cell is permitted to interact with adjacent cells or with ECM ligands will affect cell morphology and cytoskeletal organization, but may also activate β‐catenin and ERK phosphorylation events that are known regulators of gene expression and downstream biosynthesis. While cell morphology, cytoskeletal reorganization, ion channel activation, and cell‐ligand mediated events are linked in many cell types, the compelling differences shown between NP and AF cells within a single IVD structure motivate studies to reveal the precise mechanisms that govern mechanobiology in these systems.

In the process of performing a comprehensive review of the literature, we have identified some gaps in knowledge regarding topics related to mechanobiology of the IVD. Here, we briefly summarize key gaps, with the hope that future studies will inform some of the open questions in the field:

### 6.1 Development and aging

- Mechanical factors that drive phenotypic changes are not well understood in the developing, adult and aging IVD. There is a need for in vitro and ex vivo studies that identify relationships between magnitudes of spinal motion and loading conditions in vivo with long‐term changes in cellular morphology, phenotype, and metabolism.
- Tracing of cell fate through the continuum of notochord to chondrocyte‐like phenotype is needed to confirm standing hypotheses about phenotypic transition and/or transformation with aging. Nevertheless, the use of cells from younger specimens are better suited for studying early events that can lead to degeneration, whereas cells from older specimens give insight into how aged or degenerate cells react to further damage and inflammation.

### 6.2 Response of cells to loading and microenvironmental changes

- Overall, little is known about the mechanisms that govern response of cells to loading and changes in physiochemical microenvironments.
- While cell response to loading could in part be attributed to transport of chemokines like growth factors, activation of mechanoreceptors, related ion channel activation, and cytoskeletal reorganization, detailed mechanistic studies are needed to assess first‐order mechanisms. These specific signaling mechanisms that contribute to the observed anabolic and catabolic mechanobiological responses of NP and AF cells have been little studied in the context of degenerated IVDs.
- The response to altered osmolality, pH, and oxygen tension is regulated at the transcriptional level, yet the exact mechanisms by which microenvironmental changes (alone and in combinations) modulate IVD cell fate, metabolism, and interactions with immune cell infiltrates are not known.
- Knowledge of the effects of dynamic and static loading upon nutrient transport to varying compartments and substructures of the IVD. Modern approaches to monitoring tracers and contrast agents could be employed to better track transport and fluid flow to varying substructures and thus to varying cell types.

### 6.3 Cell‐ECM interactions and cell‐cell signaling

- Future studies are required to verify the precise role of ligand interactions with integrins on NP and AF cells, and to confirm a role for ligand density and specificity in activating cell signaling through known and to be discovered pathways. Emphasis on changes in cell‐ECM interactions with growth factor and chemokine signaling is also warranted.
- Cellular interactions with ECM, both visualization of and knowledge of the biochemical nature of these interactions, is needed. The role of integrin and nonintegrin matrix protein receptors, aquaporins, ion channels, and mechanosensitive channels in interacting with ECM are only limitedly studied.
- Investigations on changes in growth factor (eg, TGF‐β) activation with disc aging and changes in ECM stiffness may provide further insight into disease‐related changes.
- The mechanism by which substrate stiffness information is transduced in IVD cells is not completely understood, but it likely relates to cytoskeletal organization and related transcriptional activators.
- Given the close relationship between morphological confirmation and cell signaling through cell‐cell interactions, more work will be needed to isolate factors of cell morphology from cell‐cell mediated signaling in relationship to IVD cell phenotype.
- There is a need for in vitro culture environments that can reproduce the anaerobic metabolism and cell‐ECM interactions of the native tissue to address some of these gaps.

### 6.4 Mechanotransduction

- Only a limited number of studies have investigated ion channels, including mechanosensitive channels, and their role in IVD mechanotransduction. Investigations into the link between mechanosensitive channels, cell mechanics, and downstream transduction pathways will provide insight into physiological disc cell function.
- Evidence suggests that alternative mechanotransduction pathways mediate responses of degenerate cells to loading. Comprehensive approaches are needed to characterize and delineate alternative mechanisms of mechanotransduction with degeneration.
- Cell stress response including role of mitochondrial function in recovery from or protection against overloading are also needed.

### 6.5 Organ‐level interactions

- Knowledge of the cross talk between IVD substructures is not well‐described, particularly cross talk between the vertebral body, EP, and AF or NP. Aging, or a lifetime of spinal motion and loading, is often associated with vertebral body changes that can include EP calcification, EP thinning, or evidence of bony microdamage. Factors that drive osteoblast and osteoclast remodeling in the vertebral body, and the formation of osteophytes where relevant, would be important to determine.
- The mechanism(s) underlying the biochemical changes of the cartilage EP in disc degeneration are currently unknown, though some recent studies are beginning to address these questions^175^, ^194^. Further investigations are needed to assess related mechanism and to unveil effect of cartilage EP changes on cell mechanobiology, mediated through the cytoskeleton and other pathways. Changes in structure and cell mechanics could help in understanding the induction of mineralization, reduction in diffusion and onset of tissue damage that could result in herniation through the EP.
- Other connective tissues in the spine are also mechanosensitive and warrant consideration, including cartilage EP, vertebrae‐IVD interactions, and spinal ligaments.

To conclude, it is expected that mechanistic studies of mechanobiology of IVD cells in healthy and disease conditions may lead to novel concepts, and potential targets, for mitigating or reversing the effects of altered biomechanics on biological functions of IVD cells. Such findings will expand the basic understanding and drive the development of therapeutic strategies for recovering “physiological” mechanotransduction of the disc.
